# Low energy consumption form of the U-shaped plan office building in the Yangtze River Delta

**DOI:** 10.1038/s41598-023-38279-3

**Published:** 2023-07-12

**Authors:** Xiaoyu Ying, Fanyu Huangfu, Chi Tao

**Affiliations:** 1 Department of Architecture, Hangzhou City University, Hangzhou, 310015 China; 2 Department of Architecture, Zhejiang University, Hangzhou, 310058 China; 3 Department of Data Science and Big Data Technology, Hangzhou City University, Hangzhou, 310015 China

**Keywords:** Civil engineering, Sustainability

## Abstract

The significance of form in green building design is well recognized, as it has a substantial impact on both energy performance and construction cost. This study investigates the impact of the U-shaped plan on the energy demand, which can be flexibly applied to irregular site forms and clustered building blocks, and is widely used in existing office buildings. Specifically, we select the U-shaped plan as the object of study and collect related case data primarily from Hangzhou and Shanghai. Using Python, we generate plans based on two lengths, two angles, and depth, which serve as basic parameters and boundary conditions. The EnergyPlus platform is utilized for dynamic simulation of the energy consumption of each model, and correlation analysis using the Spearman coefficient method identifies two planar factors (depth-length ratio and modified length) that have the most significant influence on energy consumption, including five basic parameters. Regression fitting and regression evaluation of the model further identify morphological critical information in the plan. Our research reveals that the critical interval of depth-length ratio (M1) is in (0.48, 1.02), and the critical point of the modified length (M2) is 109.38 m. Furthermore, the contour plot of M1–M2–E1 is obtained, illustrating the mutual trend between morphological parameters (M1 and M2) and annual energy use intensity, which serves as a useful aid for designing low energy consumption solutions.

## Introduction

Concerns about natural resources have a long history, dating back to Thomas Malthus's "Essay on the Principle of Population" in 1798^1^. However, it was not until the 1970s that energy conservation became a focus due to events like the oil crisis and growing awareness of global warming^2^. Notably, the Paris Agreement was established during the World Climate Conference in Paris in November 2015, aiming to address climate change. China, as a significant player, set a goal to peak its CO_2_ emissions around 2030 and achieve carbon neutrality by 2060^3,4^.

The latest report from the Intergovernmental Panel on Climate Change (IPCC) highlights the substantial global mitigation potential of the buildings and construction industry for achieving the Paris Agreement goals^5^. Consequently, building energy efficiency has become a research priority in the construction sector. Building design, service design and performance, and human behavior are the three main factors that influence building energy consumption, and building design plays a critical role in energy efficiency^6^. The study of the "Simulation to Application" has shown that over 40% of the potential for building energy efficiency can be achieved during the planning and design phase^7,8^. Moreover, Pieter de Wilde, an authoritative expert in Belgium, has found that among the 303 green building technologies applied, 57% of technical measures needed to achieve green building technologies must be implemented during the planning and schematic design stage^9,10^. Thus, emphasis is increasingly placed on the design phase for building performance optimization. But, the current process of designing a building's plan is typically subjective and lacks a practical basis, resulting in lost opportunities for building energy conservation. In fact, architectural plan optimization, as an approach, can help explore more possibilities in the design stage to achieve low carbon and energy-saving objectives^11,12^.

In the context of China, architects have employed a strategy of reducing the building shape coefficient to minimize energy consumption^13^. However, in the hot summer and cold winter regions of China, the shape coefficient of office buildings has a positive correlation with the potential for using natural energy and a negative correlation with building energy consumption^14^. Although the General Specification for Energy Conservation and Renewable Energy Utilization in Buildings has provided specific requirements for the shape coefficient of public buildings in cold and severe regions^15^, it does not contain specifications for this parameter in the hot-summer and cold-winter areas. Furthermore, other factors such as plan scale, building height, plan form, and roof form have been found to have a high correlation with energy consumption^16^. As the building plan is a critical component of the schematic design that determines building dimensions and envelope orientation, it has a significant impact on construction cost, operational energy consumption, and aesthetic effects^17,18^. Building form optimization has been a subject of previous research, with studies investigating the relationship between different building plans and energy consumption^19^. For instance, in Kuwait, the aspect ratios of the zigzag, L-shaped plan, U-shaped plan, and H-shaped plan were considered to assess the impact of office building form on energy efficiency^20^. Among these plans, the U-shaped plan has been found to increase natural views, provide more light and ventilation, maximize space utilization, and reduce heat loss, making it a popular choice in the Yangtze River Delta. Nonetheless, existing studies on the plan have been limited to a single form, without examining the necessary parameters generated by the U-shaped plan and the impact of different forms resulting from the variation of its length and angle on energy consumption.

The paper study the correlation between the building form and energy consumption, take the U-shaped plan as an example. Firstly, 283 U-shaped planar models are created by determining the key parameters of the morphology through geometric principles and Python. Secondly, the EnergyPlus is conducted to dynamic simulations of the energy consumption of each model to get the annual energy use intensity (E1) and the cooling energy use intensity (E2) data. Then, spearman correlation analysis reveals that the depth-length ratio (M1) and the modified length (M2) significantly affect energy consumption. Finally, using the way of multidimensional regression fitting and evaluation obtains a graphical representation of the relationship between the U-shaped plan and energy consumption.

The novelty of the paper is a way to combine data with form. Parametric morphological description methods, data mining and correlation analysis generate, measure, and filter lots of variables, and then, through Python, investigate the relationship between variables and energy consumption, as well as visualize the results of the study. This study presents the development of energy-saving strategies that offer valuable guidance for architects during the initial design phase. The structure of the paper is as follows: The research data source and form analysis are introduced in “Methodology”. The data analysis and results are illustrated in "Analysis and results". The main conclusions are provided in "Conclusion". The limitation and future work are discussed in "Limitation and future work".

## Methodology

**Figure 1:**
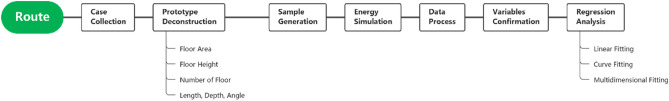
Research route.

The research process involves in developing a low-energy form for U-shaped office buildings is illustrated in Fig. 1. We collect the commonly used dimensions of the U-shaped plan in the Yangtze River Delta and conduct geometric analysis to obtain the necessary parameters for form control. The 3D model of the building is created by combining the mathematical model of the office building layout and establishing a model library. Dynamic hour-by-hour simulation of energy consumption is performed using EnergyPlus, and the corresponding energy consumption data for the 3D model is recorded. We then conduct Spearman correlation analysis and Data Mining to identify the two parameters with the highest correlation with energy consumption. Multiple regression and interpolation techniques are applied to obtain the functional relationship between the two parameters and energy consumption and to determine the mapping relationship between the building plan and energy consumption.

### Case collection

The U-shaped plan in this paper is a form with two angles at constant depth, including various forms, which are collectively referred to as the U-shaped plan due to its representativeness and extensiveness. Ten U-shaped office buildings in the Yangtze River Delta are selected as research samples from architectural websites such as archidaily, gooood, as well as Google Maps. The recorded parameters in this study encompass the plan area, length, depth, number of floors, floor height, and planar angle. Specifically, the depth of the plan is denoted as D1, while the edge lengths of the plane are categorized as follows: L1 represents the downward-facing left side, L2 represents the downward-facing middle section, L3 represents the downward-facing right side, L4 represents the upward-facing right side, L5 represents the upward-facing middle section, and L6 represents the upward-facing right side. Additionally, the angles of the plan are denoted as α1 for the left side turning angle and α2 for the right side turning angle. Table 1 summarizes the results which indicate that the U-shaped floor area ranges from 1000 to 2000 m^2^, and the length ranges from 10 to 90 m. The depth after turning is mostly similar to the depth before turning, and ignoring depth changes, the depth primarily ranges from 10 to 30 m. The number of floors ranges from 4 to 17, the building height ranges from 16 to 58 m, and the angle ranges from 60 to 300°.

**Table 1:** 10 examples of U-shaped office building plan data in the Yangtze River Delta.

Form	No	Area/m^2^	D1/m	(L1, L2, L3, L4, L5, L6)/m	α1/°	α2/°	Height/m	Number of floors
	1	1900	24	46, 86, 42, 22, 36, 18	85	90	51	17
	2	1525	15	34, 17, 31, 46, 25, 42	235	225	48	12
	3	1015	15	25, 33, 30,15, 19, 25	115	145	20	6
	4	2910	18	29, 57, 41, 43, 88,59	255	270	21	6
	5	1428	18	37, 30, 15, 21, 18, 15	90	200	28	8
	6	2154	18	48, 17, 54, 62, 50, 72	260	270	36	9
	7	1359	19	30, 25, 31, 15, 20, 14	135	130	18	4
	8	3035	23	30, 25, 45, 57, 71, 71	270	270	36	10
	9	3756	26	72, 68, 68, 51, 20, 42	100	90	21	6
	10	1881	8	36, 80, 47, 23, 48, 34	90	90	2	8

No.1 is Hangzhou Zancheng Center, No.2 is Hangzhou Water Affair Group Company Ltd. Property Management Branch, No.3 is Hangzhou Fenghuang Creative Mansion, No.4 is Zhejiang Aeronautics Industry Study Research Cooperation Base, No.5 is Zhejiang Hygiene & Health Monitoring and Evaluation Center, No.6 is Shanghai Yidian Office Complex, No.7 is Shanghai Hecheng Renovation, No.8 is Shanghai Champion Center, No.9 is Nanjing Hongfeng Technology Park, Building A1, No.10 is Xinsu Group R&D Center.

In the process of designing a building scheme, certain essential parameters must be determined, such as the building's plan form, dimensions, and floor height. In this study, our focus is on the building plan. To that end, we quantified the building area, number of floors, and floor height. Combining the collected case data, we set the building area at 1800 m^2^, the total number of floors at 10, the height of each floor at 3.6 m, the total building height at 36 m, and the total building area at 18,000 m^2^.

### Prototype deconstruction

**Figure 2:**
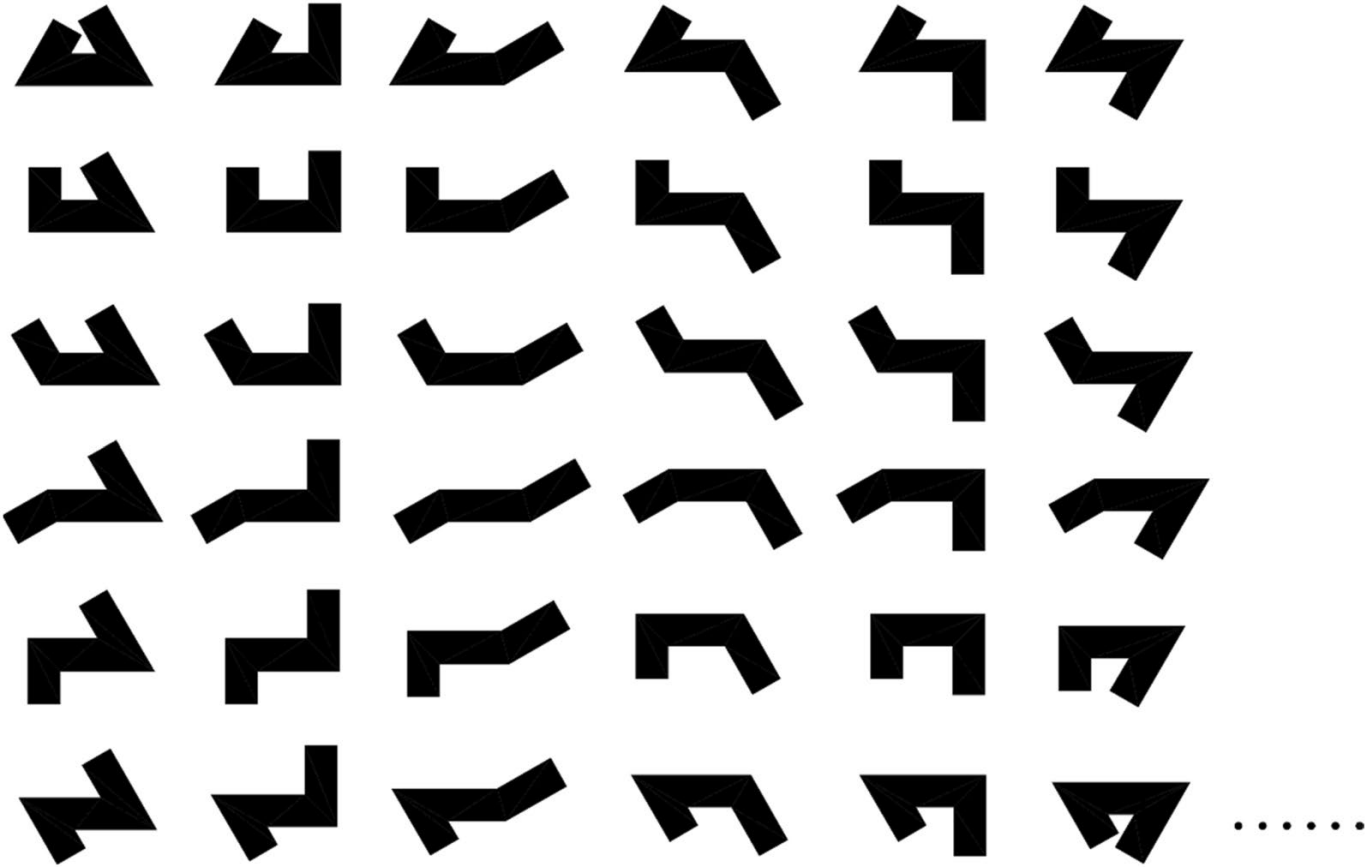
Different forms of the U-shaped plan.

Rectangular plan is the most prevalent type of office building. However, due to site-specific requirements such as site shape, building combination, functional division, and aesthetic form, different plans are derived from the rectangular plan. One plan is the U-shaped plan, which can adapt to different sites by altering lengths and angles to create diverse spatial arrangements, as illustrated in Fig. 2. In addition, it enhances indoor air quality, lighting and natural ventilation improve indoor comfort. The surrounding open space created by the U-shaped plan provides a tranquil outdoor environment with natural scenery, which makes it a popular choice in practice. In this paper, we examine the relationship between building planning and energy consumption, taking the U-shaped plan as a case study.

**Figure 3:**
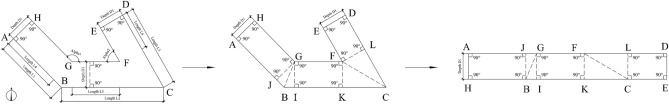
The U-shaped plan parameterization.

The parameterization of the U-shaped plan in office buildings establishes a connection between form and numbers through geometric analysis. To analyze and generate the plan in a systematic, comprehensive, and efficient manner, computer language is applied. Figure 3 illustrates the starting point at point A, with line segments extending counterclockwise to points B, C, D, E, F, G, and H to form a closed U-shaped plan. The orientation of the building is north–south, with L1 as the AB line segment, L2 as the BC line segment, L3 as the CD line segment, L4 as the GH line segment, L5 as the FG line segment, L6 as the EF line segment, D1 as the line segment AH and line segment DE, α1 as the angle ∠FGH, and α2 as the angle ∠EFG. Two pairs of right triangles (Rt△BIG ≌ Rt△BJG and Rt△CKF ≌ Rt△CLF) are respectively equal, indicating that trapezoid ABGH and trapezoid CDEF in the U-shaped plan can be reassembled with trapezoid BCFG by flipping to form a rectangular plan with the same area. This feature of the U-shaped plan enables the determination of necessary parameters of its plan, including D1, α1, α2, L1, and L3, where L4 and L6, L1 and L5, L3 and L5, and L2 and L4, L2 and L6 can replace L1 and L3.

The boundary conditions of building model parameters are derived from the data collected from the cases, as presented in Table 2. These parameters are incorporated into the algorithm to generate a building model that closely resembles the actual case. During the experiment, when the area of building's standard floor is known, five essential parameters are required based on the geometric analysis. The mathematical model of the building floor plan model is then generated on the Python platform using these parameters, producing the 283 group U-shaped floor plan of the study object.

**Table 2:** Boundary conditions.

Type (unit)	Range	Step
Area (m^2^)	1800	0
D1 (m)	10–30	10
L1, L3 (m)	10–90	30
α1, α2 (°)	60–300	30

### Energy simulation and data process

**Figure 4:**
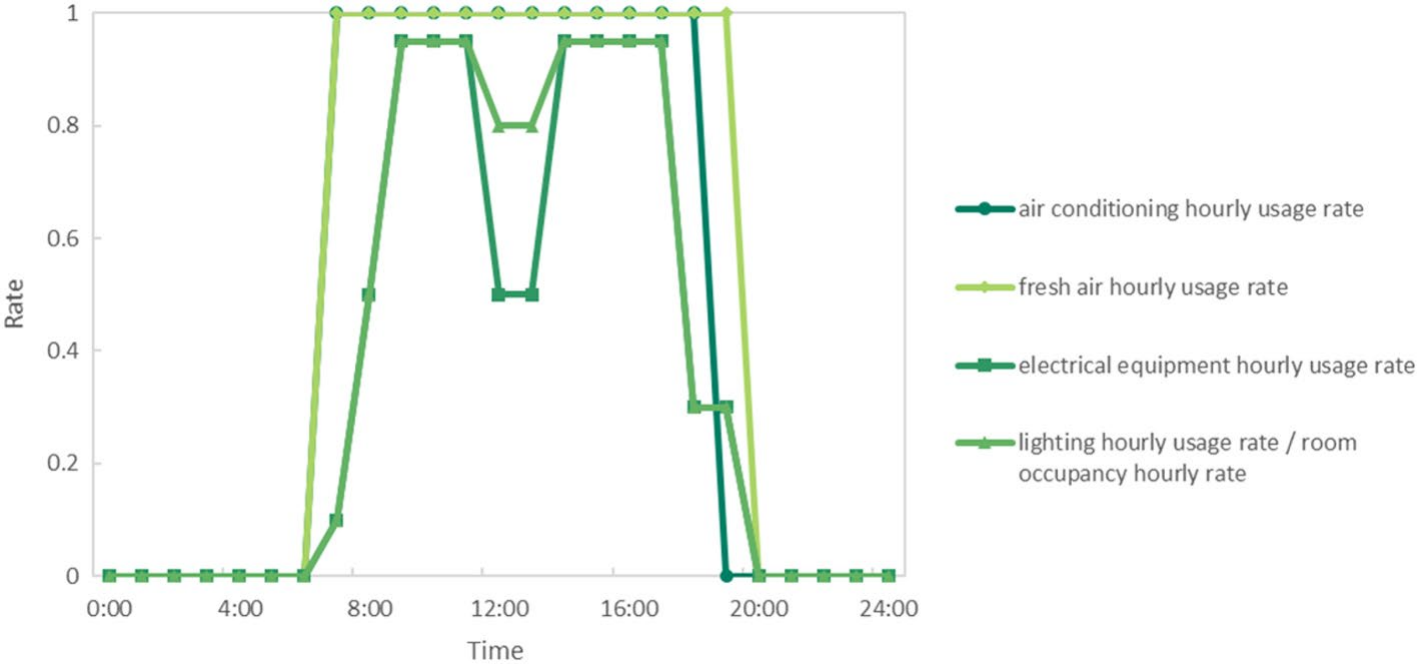
Equipment rate.

The research focused on the office buildings and utilized the requirements for its work and rest outlined in specification^10^ to set up the simulation, which included factors such as air conditioning, lighting, fresh air system, electrical equipment hourly utilization rate, and personnel presence rate. Figure 4 illustrates these parameters.

The study follows the guidelines set in specification^10^ to establish the parameters for energy consumption simulation, detailed in Table 3. The simulation is set up for an office building located in Hangzhou City, Zhejiang Province, with 10 floors, each with a floor height of 3.6 m and a total building height of 36 m. The floor area is 1800 m^2^ with a window-to-wall ratio of 0.3, window sill height of 0.8 m, window height of 1.5 m, and window width of 1.2 m. The air conditioning system adopts fan coils and a fresh air system with an air-cooled chiller, where the cooling coefficient COP is 3.00 and the gas boiler serves as the heat source with a thermal efficiency of 0.92. Cooling starts at 28 °C and lowers to 26 °C, while heating starts at 12 °C and rises to 20 °C. The average fresh air volume is 30 m^3^·(h p)^−1^. The working and resting requirements for the office building in specification^10^ are adopted, with a total of 14 days of annual leave and Monday to Friday designated as working days.

**Table 3:** Parameters' condition.

Architectural model parameters		Building interior parameters	
Building type	Office building	Cooling temperature	28 °C → 26 °C
Simulation site	Hangzhou, Zhejiang Province	Heating temperature	12 °C → 20 °C
Floor height	3.6 m	Room dehumidification	90% → 40%
Number of floors	10	Domestic hot water	0.2 L·(m^2^)^−1^-day
Window-to-wall ratio	0.3	Equipment cooling	15 W (m^2^)^−1^
Total building height	36 m	Personnel density	0.111 p (m^2^)^−1^
Standard floor area	1200 m^2^	Target illumination	300 lx
Total building area	12,000 m^2^	Lighting power density	8 W m^2^)^−1^

Studying the relationship between physical objects and energy consumption poses challenges due to peculiarities such as long construction cycles, significant individual variability, and diverse use cycles. To overcome these challenges, the EnergyPlus software simulation is utilized. The simulation requires building parameters such as D1 for depth, L1 and L3 for length, α1 and α2 for angles, as well as E1 for annual energy use intensity and E2 for annual cooling energy use intensity. The modelling and simulation lead to the acquisition of Appendix A.

To avoid complexity and limitations in the control of the building's plan, we perform data mining and filtering on the five parameters (L1, L3, α1, α2, and D1) related to the floor area. This approach generates new variables and reduces the number of planar parameters to study their coupled effects on the plan. Although the five original parameters have controlled the plan in practice, their information is insufficient and not very meaningful for analyzing length changes in different floor areas or the changing pattern of energy consumption for different angles.

The study employed the Spearman coefficient method to perform correlation analysis^17^. The formula used for calculating the coefficient is expressed as follows:

1 $$\begin{array}{c}\rho =1-\frac{6\sum {d}_{i}^{2}}{n\left({n}^{2}-1\right)}\end{array}$$

**Figure 5:**
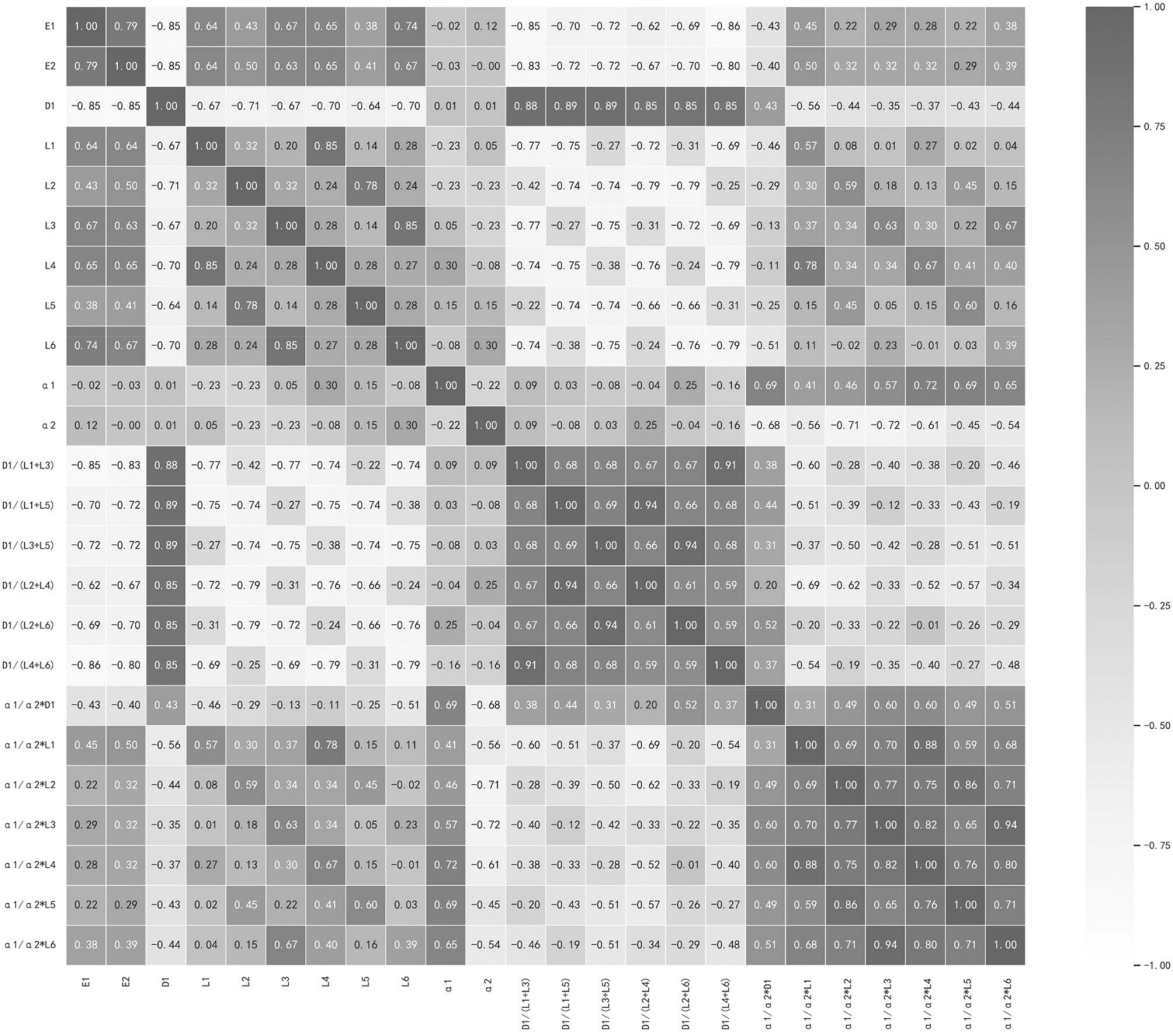
Correlation analysis.

Here, ρ denotes the correlation coefficient, n represents the sample size, and di stands for the difference in rank between the two variables. Figure 5 presents the correlation results between variables after applying the aforementioned data processing method. From the numerous derived parameters, two variables, namely the D1/(L1 + L3) depth-length ration, and α1/α2 × L1 modified length, are identified for energy consumption research in building design.

Based on the prototype deconstruction outlined in “Prototype deconstruction”, the identification of five fundamental parameters (L1, L3, α1, α2, and D1) is necessary to determine the unique plan when the plan's area is established. However, these basic parameters offer limited and biased information. For instance, in architectural design, the floor area varies across different projects, rendering the study of length parameters less meaningful. Additionally, the energy consumption patterns associated with different angles under varying length parameters may not exhibit obvious trends. Consequently, the original base parameters undergo processing to generate new variables for data mining and screening purposes. Among the numerous newly derived parameters, two variables, namely M1 (D1/(L1 + L3)) and M2 (α1/α2 × L1), are selected for investigating the U-shape. Notably, M1 and M2 exert a more substantial influence on annual energy use intensity and cooling energy use intensity compared to other generated variables of the same character. The correlation coefficients between M1 and E1, M1 and E2, M2 and E1, and M2 and E2 are found to be 0.85, 0.83, 0.45, and 0.5, respectively. Furthermore, upon analyzing the U-shaped corresponding to M1, it becomes apparent that it governs the proportion of heat gained by the U-shaped plane, rather than relying on specific side length values. Similarly, M2 regulates the ratio between the two angles, controlling the plane's twisting direction. Meanwhile, these variables encompass the five basic parameters required to determine the U-shaped plan.

## Analysis and results

In this paper, we study the relationship between the U-shaped plan depth-length ratio (M1), modified length (M2), annual energy use intensity (E1), and annual cooling energy use intensity (E2) using regression analysis. Our two-dimensional regression fitting analysis examines the relationships about M1–E1, M1–E2, M2–E1, and M2–E2. In addition, we conducted a three-dimensional regression fitting analysis to investigate the relationships about M1–M2–E1 and M1–M2–E2. Finally, we evaluated our results using regression index. These findings provide important insights into the relationships between U-shaped plan design factors and energy use intensity in buildings.

### Two-dimensional fitting and evaluation

**Figure 6:**
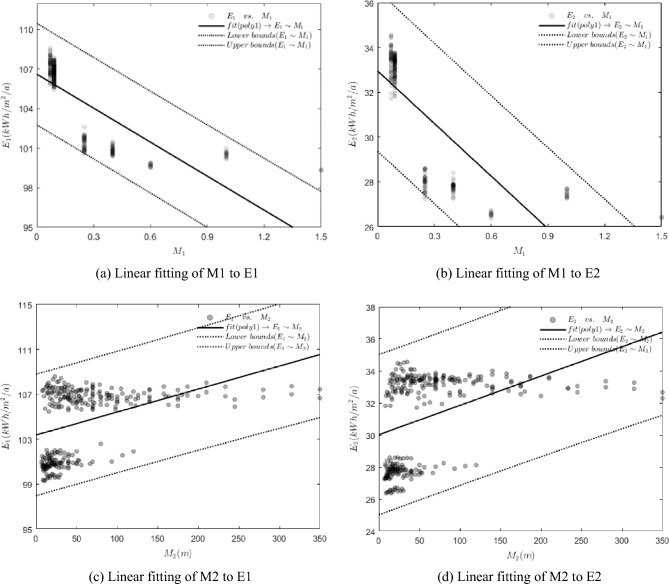
Linear fitting of the depth-length ratio (M1) and the modified length (M2) to the annual energy use intensity and cooling energy use intensity.

In this study, the relationship between the depth-to-length ratio (M1) and modified length (M2) of buildings with their annual energy intensity (E1) and cooling energy intensity (E2) was examined through scatter plots and linear fitting. Specifically, In Fig. 6a,b, the scatter plots depict the relationship between the depth-to-length ratio (M1) and the annual energy intensity (E1) and cooling energy intensity (E2), respectively. Similarly, Fig. 6c,d show the scatter plots of the modified length (M2) versus annual energy intensity (E1) and cooling energy intensity (E2), respectively. The first fit analysis reveals an inverse proportionality between M1 and both E1 and E2, indicating a decrease in annual energy use intensity and cooling energy use intensity with an increase in building depth-to-length ratio. In contrast, both E1 and E2 show a positive proportionality with M2, suggesting an increase in annual energy use intensity and cooling energy use intensity with an increase in building modified length. Although the scatter plot indicates the trend between the variables and energy use, it is apparent that there is a substantial gap between the linear fit and the trend of the scatter plot in an underfitting state. Therefore, a polynomial fit analysis is performed in the subsequent analysis.

**Figure 7:**
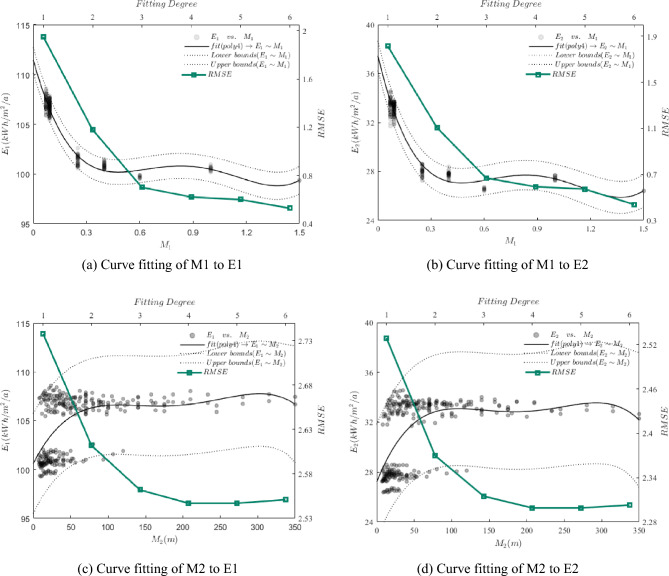
Curve fitting of the depth-length ratio (M1) and the modified length (M2) to the annual energy use intensity and cooling energy use intensity.

The regression model for M1–E1 and M1–E2 was developed through six polynomial fits, and the fourth fit was found to have the smoothest root mean square error. Figure 7a,b illustrate the fit results. The curves show that M1 within the interval (0, 0.48) has monotonically decreasing values for E. The curves show that M1 within the interval (0, 0.47) has monotonically decreasing values for E2. For the interval (0.48, 1.5), E1 remain in a flat phase with small changes. For the interval (0.47, 1.5), E2 remain in a flat phase with small changes. At M1 = 1.37, E1 reaches a minimum value of 98.81 kWh/m^2^/a, while E2 reaches a minimum value of 25.88 kWh/m^2^/a. Based on these findings, we can conclude that: (1) the annual energy use intensity and cooling energy use intensity of buildings decrease significantly with the increase of the depth-length ratio of the U-shaped plan when it falls within the interval (0, 0.47); (2) the annual energy use intensity of the building does not change significantly with the depth-length ratio of the building when it is greater than 0.48, and the energy consumption value is relatively low; (3) the minimum point of annual energy consumption for M1 of the U-shaped plan in the interval (0, 1.5) is (1.37, 98.81), indicating that the minimum annual energy consumption of 98.81 kWh/m^2^/a can be achieved when the depth-length ratio is 1.37. The minimum point of cooling energy consumption is also (1.37, 25.88), implying that the minimum annual energy consumption of 25.88 kWh/m^2^/a can be obtained when the depth-length ratio is 1.37.

Following six polynomial fits of M2–E1 and M2–E2, we have discovered the regression model that results in smoothing of the root mean square error by the fourth fit. The fit results are shown in Fig. 7c,d, indicating that the value of E1 increases monotonically for M2 in the interval of (0, 114) while the value of E2 increases monotonically for M2 in the interval of (0, 109.38). Moreover, E1 is in the smoothing stage for M2 in the interval of (114, 350), and E2 is in the smoothing stage for M2 in the interval of (109.38, 350). The study's findings allow us to draw the following conclusions: (1) M2 in the interval of (0, 114), the correction length of U-shaped plan significantly increases the annual energy intensity of the building; M2 in the interval of (0, 109.38), the correction length of U-shaped plan significantly increases the cooling energy intensity of the building. (2) When the modified length of the U-shaped plan is within the interval of (114, 350) and (109.38, 350), the annual energy use intensity and cooling energy use intensity of the building system varies less in comparison to the interval (0, 114) and (0, 109.38). (3) The peak annual energy use within the range of (0, 350) occurs at a modified length of U-shaped plan of 302.5 m with an annual cooling energy use of 107.76 kWh/m^2^/a, while the highest cooling energy use of 33.55 kWh/m^2^/a occurs at a modified length of U-shaped plan of 292.6 m.

### Three-dimensional fitting and evaluation

"Two-dimensional fitting and evaluation" presents an analysis of the individual variables' effects on energy consumption, drawing conclusions on the impact of the depth-length ratio and modified length, resulting in improved regression models. Furthermore, to investigate the impact of the two variables on energy consumption simultaneously, three-dimensional regression models and contour plots for M1–M2–E1 and M1–M2–E2 are developed. These models enable the observation of the joint effect of the two variables on energy consumption. By comparing the two-dimensional and three-dimensional fitting results, the similarities and differences in the effects of the depth-length ratio and modified length on energy consumption are verified. The generated contour diagrams are practical references for architects when optimizing building plan design.

**Figure 8:**
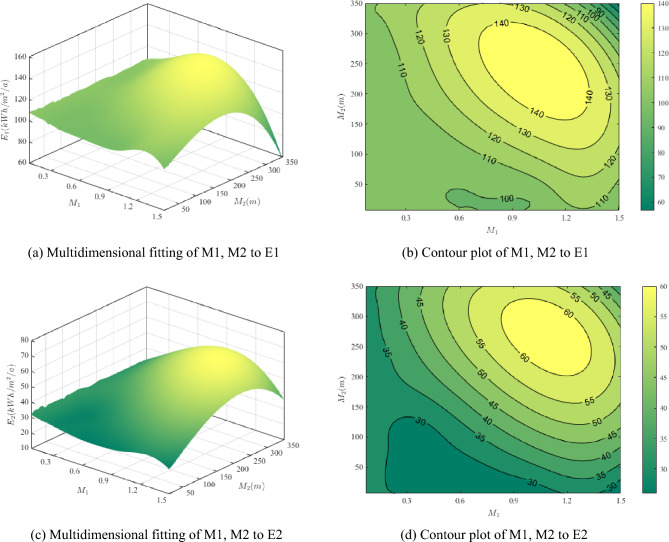
Multidimensional fitting of the depth-length ratio (M1) and the modified length (M2) to the annual energy use intensity and cooling energy use intensity.

"Three-dimensional fitting and evaluation" presents the results of the interpolation fit for M1, M2, and E1, and the three-dimensional fitting results for M1, M2, and E2. The contour plots are used to visually illustrate the significance of the interaction between the two variables. The circle indicates that the interaction between the two factors is not significant, while the ellipse indicates the opposite. In Fig. 8b,d, the height lines are elliptical, indicating that the interaction between the two factors is significant. Figure 8a,c respectively show the fitting results for E1 and E2, where the coefficient of determination (R^2^) is 0.99 (M1–M2–E1) and 0.994 (M1–M2–E2), the error sum of squares (SSE) is 16.854 (M1–M2–E1) and 9.685 (M1–M2–E2), and the root mean squared error (RMSE) is 0.68 (M1–M2–E1) and 0.6 (M1–M2–E2). The fitting effect and validation are deemed to be good. Both models are presented as a smooth surface in space, which is mapped to a plan with a vortex to highlight the region of high energy consumption, as shown by the yellow vortex kernel in Fig. 8b,d. This is not conducive to low-energy consumption of the U-shaped plan. It is also observed that energy consumption fluctuates more when M1 is at (1.02, 1.5) and M2 is at (247.2, 350), which is not conducive to control. In addition, the trend of another value under the low energy consumption target can be observed for any M1 value or M2 value. For example, when M1 equals 1, indicating that the length of the U-shaped plan depth is the same as the sum of the sizes of the two arm sides, a lower year-round energy intensity can be achieved if M2 is far away from 247.1 m, and the value becomes lower the farther away M2 is.

## Conclusion

In this investigation, we examined the impact of morphology on building energy consumption by simulating energy consumption of 283 different U-shaped plans with varying depth, lengths, and angles. Using the Spearman coefficient formula to select the two highest morphological parameters, the correlation is obtained between the U-shaped plan and energy consumption. We utilized regression fitting and regression evaluation indexes to examine the impact of the two variables on energy consumption, and identified the critical point of morphological parameters under the low energy consumption target, the optimal morphological value, and the contour plot of the impact of the two variables on energy consumption simultaneously. This study is unique in its combination of data mining and correlation analysis to generate, measure, and filter the variables. Furthermore, it introduced a parametric morphological description method and utilized the Python computing platform to successfully combine data with morphology through data visualization. The methodology introduced in this study has the potential to be extended to many other forms of architecture.

The U-shaped building scheme's specific conclusions are as follows. First, for a single variable's effect on energy consumption, M1 values between 0 and 0.48 result in lower energy consumption intensity with increasing M1 values, and the effect is evident. Similarly, M2 values less than 109.38 result in lower energy consumption intensity with decreasing M2 values. Second, examining contour Fig. 8b reveals that coupling depth-length ratio (M1) and correction length (M2) with energy consumption produces peaks in both M1 and M2. Specifically, energy consumption is higher the closer M1 is to 1.02, and the closer M2 is to 247.1 m. Finally, fitting methods showed that the optimal U-shaped building scheme for the Yangtze River Delta region reduces energy consumption by maintaining a depth-length ratio between 0.48 and 1.02 and modified length below 109.38 m.

**Figure 9:**
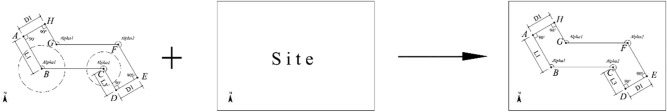
Application demonstration of the U-shaped plan.

Figure 9 illustrates that the directions of the L1 and L3 line segments in the U-shaped plan are determined based on the building direction, site shape, landscape direction, and space combination, with the two ends of the L2 line segment as the origin of a circle. Subsequently, the depth of the D1 line segments is established based on the practical case. By selecting an appropriate length value of L1 based on the recommendation that M2 should be less than 109.38 m, the length value of L3 can be calculated according to the M1 interval (0.48, 1.02) that meets actual requirements to form the outer contour of the building plan. This approach allows for U-shaped buildings to reduce energy consumption without limiting angle, length, or plan form, while also saving time and effort in simulation software, thus offering a new avenue for energy-saving building design.

The contour map depicting the energy consumption of the U-shaped plan in the Yangtze River Delta region, as affected by the depth-length ratio and modified length, serves as a visual tool for selecting a low-energy floor plan. Nonetheless, during the architectural design process, careful consideration must be given to factors such as the suitability of the floor plan to the site shape, internal functional layout requirements, and façade design.

## Limitation and future work

Further progress in this study will enable the exploration of various aspects, including the advancement of augmentation of data volume, empirical investigations and validation of theoretical models. Additionally, analyzing building shapes as variables while adhering to consistent constraints would facilitate the examination of shape-related trends across different regions. This approach can be extended to incorporate additional constraints beyond those considered in this research, such as building orientation, window-to-wall ratio, and insulation material, among others. To enhance the study's efficiency and usability, future endeavors will encompass the development of a user-friendly interface, enabling architects and design teams to effortlessly utilize the model. This interface will aid the design team in minimizing energy consumption and enhancing the overall quality of the constructed environment.

## Supplementary Information

Supplementary Information.

## Data Availability

All data generated or analysed during this study are included in this published article [and its supplementary information files].
