# Supplementary material for: Low energy consumption form of the U-shaped plan office building in the Yangtze River Delta
Source: Sci Rep. 2023 Jul 12;13:11250. doi: 10.1038/s41598-023-38279-3 (PMC10338471; doi:10.1038/s41598-023-38279-3)
Supplement: Supplementary file 1 — Supplementary Information. [file 41598_2023_38279_MOESM1_ESM.pdf]

Appendix A

No.	D1 /m	L1 /m	L2 /m	L3 /m	L5 /m	L4 /m	L6 /m	α1 /°	α2 /°	E1 /kWh·(m²·a)$^{-1}$	E2 /kWh·(m²·a)$^{-1}$
1	10	40	90.00	70	70	22.68	67.32	60	150	105.46	32.21
2	10	40	84.64	70	70	22.68	72.68	60	210	105.72	32.48
3	10	40	81.55	70	70	22.68	75.77	60	240	106.83	32.68
4	10	40	77.32	70	70	22.68	80.00	60	270	107.38	32.45
5	10	40	70.00	70	70	22.68	87.32	60	300	107.32	31.82
6	10	40	90.00	70	70	30.00	60.00	90	90	106.82	32.75
7	10	40	85.77	70	70	30.00	64.23	90	120	106.54	33.04
8	10	40	82.68	70	70	30.00	67.32	90	150	105.86	32.92
9	10	40	77.32	70	70	30.00	72.68	90	210	106.1	33.12
10	10	40	74.23	70	70	30.00	75.77	90	240	107.18	33.36
11	10	40	70.00	70	70	30.00	80.00	90	270	107.72	33.21
12	10	40	62.68	70	70	30.00	87.32	90	300	107.59	32.59
13	10	40	85.77	70	70	34.23	60.00	120	90	106.87	33.08
14	10	40	81.55	70	70	34.23	64.23	120	120	106.61	33.39
15	10	40	78.45	70	70	34.23	67.32	120	150	105.92	33.26
16	10	40	73.09	70	70	34.23	72.68	120	210	106.15	33.43
17	10	40	70.00	70	70	34.23	75.77	120	240	107.22	33.72
18	10	40	65.77	70	70	34.23	80.00	120	270	107.72	33.54
19	10	40	58.45	70	70	34.23	87.32	120	300	107.55	32.96
20	10	40	90.00	70	70	37.32	52.68	150	60	106.27	32.47
21	10	40	82.68	70	70	37.32	60.00	150	90	106.54	33.12
22	10	40	78.45	70	70	37.32	64.23	150	120	106.29	33.42
23	10	40	75.36	70	70	37.32	67.32	150	150	105.61	33.27
24	10	40	70.00	70	70	37.32	72.68	150	210	105.82	33.4
25	10	40	66.91	70	70	37.32	75.77	150	240	106.82	33.64
26	10	40	62.68	70	70	37.32	80.00	150	270	107.29	33.48
27	10	40	55.36	70	70	37.32	87.32	150	300	107.1	32.87
28	10	40	84.64	70	70	42.68	52.68	210	60	106.32	32.6
29	10	40	77.32	70	70	42.68	60.00	210	90	106.59	33.25
30	10	40	73.09	70	70	42.68	64.23	210	120	106.33	33.53
31	10	40	70.00	70	70	42.68	67.32	210	150	105.65	33.38
32	10	40	64.64	70	70	42.68	72.68	210	210	105.76	33.38
33	10	40	61.55	70	70	42.68	75.77	210	240	106.79	33.64
34	10	40	57.32	70	70	42.68	80.00	210	270	107.24	33.44
35	10	40	50.00	70	70	42.68	87.32	210	300	107.06	32.77
36	10	40	81.55	70	70	45.77	52.68	240	60	106.79	32.76
37	10	40	74.23	70	70	45.77	60.00	240	90	107.06	33.42
38	10	40	70.00	70	70	45.77	64.23	240	120	106.81	33.73
39	10	40	66.91	70	70	45.77	67.32	240	150	106.06	33.51
40	10	40	61.55	70	70	45.77	72.68	240	210	106.18	33.51
41	10	40	58.45	70	70	45.77	75.77	240	240	107.21	33.75

42	10	40	54.23	70	70	45.77	80.00	240	270	107.69	33.59
43	10	40	46.91	70	70	45.77	87.32	240	300	107.53	32.91
44	10	40	77.32	70	70	50.00	52.68	270	60	106.96	32.71
45	10	40	70.00	70	70	50.00	60.00	270	90	107.24	33.39
46	10	40	65.77	70	70	50.00	64.23	270	120	106.93	33.62
47	10	40	62.68	70	70	50.00	67.32	270	150	106.22	33.44
48	10	40	57.32	70	70	50.00	72.68	270	210	106.33	33.43
49	10	40	54.23	70	70	50.00	75.77	270	240	107.36	33.69
50	10	40	50.00	70	70	50.00	80.00	270	270	107.86	33.57
51	10	40	42.68	70	70	50.00	87.32	270	300	107.62	32.76
52	10	40	70.00	70	70	57.32	52.68	300	60	106.85	32.44
53	10	40	62.68	70	70	57.32	60.00	300	90	107.11	33.07
54	10	40	58.45	70	70	57.32	64.23	300	120	106.82	33.33
55	10	40	55.36	70	70	57.32	67.32	300	150	106.08	33.12
56	10	40	50.00	70	70	57.32	72.68	300	210	106.2	33.1
57	10	40	46.91	70	70	57.32	75.77	300	240	107.26	33.42
58	10	40	42.68	70	70	57.32	80.00	300	270	107.69	33.24
59	10	70	90.00	40	70	52.68	37.32	60	150	105.92	32.63
60	10	70	84.64	40	70	52.68	42.68	60	210	105.97	32.73
61	10	70	81.55	40	70	52.68	45.77	60	240	106.64	32.69
62	10	70	77.32	40	70	52.68	50.00	60	270	107.03	32.34
63	10	70	70.00	40	70	52.68	57.32	60	300	107.15	31.82
64	10	70	90.00	40	70	60.00	30.00	90	90	106.98	33.36
65	10	70	85.77	40	70	60.00	34.23	90	120	106.84	33.42
66	10	70	82.68	40	70	60.00	37.32	90	150	106.47	33.31
67	10	70	77.32	40	70	60.00	42.68	90	210	106.57	33.5
68	10	70	74.23	40	70	60.00	45.77	90	240	107.22	33.49
69	10	70	70.00	40	70	60.00	50.00	90	270	107.62	33.22
70	10	70	62.68	40	70	60.00	57.32	90	300	107.63	32.65
71	10	70	85.77	40	70	64.23	30.00	120	90	106.91	33.53
72	10	70	81.55	40	70	64.23	34.23	120	120	106.79	33.6
73	10	70	78.45	40	70	64.23	37.32	120	150	106.46	33.64
74	10	70	73.09	40	70	64.23	42.68	120	210	106.56	33.7
75	10	70	70.00	40	70	64.23	45.77	120	240	107.21	33.73
76	10	70	65.77	40	70	64.23	50.00	120	270	107.55	33.4
77	10	70	58.45	40	70	64.23	57.32	120	300	107.55	32.91
78	10	70	90.00	40	70	67.32	22.68	150	60	105.98	33.04
79	10	70	82.68	40	70	67.32	30.00	150	90	106.14	33.3
80	10	70	78.45	40	70	67.32	34.23	150	120	106.05	33.44
81	10	70	75.36	40	70	67.32	37.32	150	150	105.71	33.37
82	10	70	70.00	40	70	67.32	42.68	150	210	105.81	33.4
83	10	70	66.91	40	70	67.32	45.77	150	240	106.4	33.38
84	10	70	62.68	40	70	67.32	50.00	150	270	106.71	33.1
85	10	70	55.36	40	70	67.32	57.32	150	300	106.7	32.6

86	10	70	84.64	40	70	72.68	22.68	210	60	105.9	33.04
87	10	70	77.32	40	70	72.68	30.00	210	90	106.07	33.29
88	10	70	73.09	40	70	72.68	34.23	210	120	105.97	33.43
89	10	70	70.00	40	70	72.68	37.32	210	150	105.64	33.38
90	10	70	64.64	40	70	72.68	42.68	210	210	105.65	33.28
91	10	70	61.55	40	70	72.68	45.77	210	240	106.22	33.24
92	10	70	57.32	40	70	72.68	50.00	210	270	106.53	32.92
93	10	70	50.00	40	70	72.68	57.32	210	300	106.52	32.3
94	10	70	81.55	40	70	75.77	22.68	240	60	106.73	33.36
95	10	70	74.23	40	70	75.77	30.00	240	90	106.9	33.62
96	10	70	70.00	40	70	75.77	34.23	240	120	106.81	33.77
97	10	70	66.91	40	70	75.77	37.32	240	150	106.43	33.66
98	10	70	61.55	40	70	75.77	42.68	240	210	106.43	33.55
99	10	70	58.45	40	70	75.77	45.77	240	240	107.01	33.5
100	10	70	54.23	40	70	75.77	50.00	240	270	107.3	33.13
101	10	70	46.91	40	70	75.77	57.32	240	300	107.32	32.43
102	10	70	77.32	40	70	80.00	22.68	270	60	107.01	33.27
103	10	70	70.00	40	70	80.00	30.00	270	90	107.19	33.53
104	10	70	65.77	40	70	80.00	34.23	270	120	107.04	33.61
105	10	70	62.68	40	70	80.00	37.32	270	150	106.66	33.42
106	10	70	57.32	40	70	80.00	42.68	270	210	106.68	33.42
107	10	70	54.23	40	70	80.00	45.77	270	240	107.25	33.35
108	10	70	50.00	40	70	80.00	50.00	270	270	107.55	32.96
109	10	70	42.68	40	70	80.00	57.32	270	300	107.44	32.04
110	10	70	70.00	40	70	87.32	22.68	300	60	106.67	32.66
111	10	70	62.68	40	70	87.32	30.00	300	90	106.83	32.9
112	10	70	58.45	40	70	87.32	34.23	300	120	106.71	33.04
113	10	70	55.36	40	70	87.32	37.32	300	150	106.35	32.96
114	10	70	50.00	40	70	87.32	42.68	300	210	106.36	32.74
115	10	70	46.91	40	70	87.32	45.77	300	240	106.93	32.64
116	10	70	42.68	40	70	87.32	50.00	300	270	107.09	31.94
117	10	70	67.32	70	40	52.68	60.00	60	90	106.78	31.71
118	10	70	63.09	70	40	52.68	64.23	60	120	106.76	32.56
119	10	70	60.00	70	40	52.68	67.32	60	150	106.18	32.7
120	10	70	54.64	70	40	52.68	72.68	60	210	106.47	33.36
121	10	70	51.55	70	40	52.68	75.77	60	240	107.57	33.82
122	10	70	47.32	70	40	52.68	80.00	60	270	108.03	33.77
123	10	70	40.00	70	40	52.68	87.32	60	300	107.88	33.46
124	10	70	67.32	70	40	60.00	52.68	90	60	107.5	32.46
125	10	70	60.00	70	40	60.00	60.00	90	90	107.64	33.13
126	10	70	55.77	70	40	60.00	64.23	90	120	107.38	33.52
127	10	70	52.68	70	40	60.00	67.32	90	150	106.72	33.51
128	10	70	47.32	70	40	60.00	72.68	90	210	107.03	33.98
129	10	70	44.23	70	40	60.00	75.77	90	240	108.11	34.42

130	10	70	40.00	70	40	60.00	80.00	90	270	108.58	34.48
131	10	70	32.68	70	40	60.00	87.32	90	300	108.33	34.09
132	10	70	63.09	70	40	64.23	52.68	120	60	107.38	32.86
133	10	70	55.77	70	40	64.23	60.00	120	90	107.56	33.43
134	10	70	51.55	70	40	64.23	64.23	120	120	107.29	33.76
135	10	70	48.45	70	40	64.23	67.32	120	150	106.65	33.72
136	10	70	43.09	70	40	64.23	72.68	120	210	106.97	34.08
137	10	70	40.00	70	40	64.23	75.77	120	240	108.04	34.56
138	10	70	35.77	70	40	64.23	80.00	120	270	108.43	34.52
139	10	70	28.45	70	40	64.23	87.32	120	300	108.15	34.13
140	10	70	60.00	70	40	67.32	52.68	150	60	106.54	32.55
141	10	70	52.68	70	40	67.32	60.00	150	90	106.8	33.19
142	10	70	48.45	70	40	67.32	64.23	150	120	106.56	33.53
143	10	70	45.36	70	40	67.32	67.32	150	150	105.89	33.42
144	10	70	40.00	70	40	67.32	72.68	150	210	106.21	33.69
145	10	70	36.91	70	40	67.32	75.77	150	240	107.17	34.07
146	10	70	32.68	70	40	67.32	80.00	150	270	107.58	34.09
147	10	70	25.36	70	40	67.32	87.32	150	300	107.34	33.55
148	10	70	54.64	70	40	72.68	52.68	210	60	106.54	32.66
149	10	70	47.32	70	40	72.68	60.00	210	90	106.83	33.36
150	10	70	43.09	70	40	72.68	64.23	210	120	106.57	33.64
151	10	70	40.00	70	40	72.68	67.32	210	150	105.9	33.52
152	10	70	34.64	70	40	72.68	72.68	210	210	105.97	33.45
153	10	70	31.55	70	40	72.68	75.77	210	240	106.97	33.75
154	10	70	27.32	70	40	72.68	80.00	210	270	107.39	33.55
155	10	70	20.00	70	40	72.68	87.32	210	300	107.19	32.63
156	10	70	51.55	70	40	75.77	52.68	240	60	107.41	32.97
157	10	70	44.23	70	40	75.77	60.00	240	90	107.69	33.67
158	10	70	40.00	70	40	75.77	64.23	240	120	107.45	34.01
159	10	70	36.91	70	40	75.77	67.32	240	150	106.69	33.78
160	10	70	31.55	70	40	75.77	72.68	240	210	106.73	33.67
161	10	70	28.45	70	40	75.77	75.77	240	240	107.71	33.86
162	10	70	24.23	70	40	75.77	80.00	240	270	108.05	33.52
163	10	70	16.91	70	40	75.77	87.32	240	300	107.43	31.94
164	10	70	47.32	70	40	80.00	52.68	270	60	107.72	32.88
165	10	70	40.00	70	40	80.00	60.00	270	90	108.03	33.61
166	10	70	35.77	70	40	80.00	64.23	270	120	107.69	33.83
167	10	70	32.68	70	40	80.00	67.32	270	150	106.94	33.65
168	10	70	27.32	70	40	80.00	72.68	270	210	106.93	33.44
169	10	70	24.23	70	40	80.00	75.77	270	240	107.86	33.58
170	10	70	20.00	70	40	80.00	80.00	270	270	107.9	32.86
171	10	70	40.00	70	40	87.32	52.68	300	60	107.45	32.29
172	10	70	32.68	70	40	87.32	60.00	300	90	107.68	32.93
173	10	70	28.45	70	40	87.32	64.23	300	120	107.38	33.21

174	10	70	25.36	70	40	87.32	67.32	300	150	106.63	33.03
175	10	70	20.00	70	40	87.32	72.68	300	210	106.56	32.65
176	10	70	16.91	70	40	87.32	75.77	300	240	107.1	32.47
177	20	10	59.28	10	70	15.36	15.36	210	210	100.17	27.65
178	20	10	53.09	10	70	15.36	21.55	210	240	100.33	27.46
179	20	10	44.64	10	70	15.36	30.00	210	270	100.55	27.28
180	20	10	30.00	10	70	15.36	44.64	210	300	100.97	27.25
181	20	10	53.09	10	70	21.55	15.36	240	210	100.27	27.68
182	20	10	46.91	10	70	21.55	21.55	240	240	100.42	27.49
183	20	10	38.45	10	70	21.55	30.00	240	270	100.62	27.34
184	20	10	23.81	10	70	21.55	44.64	240	300	100.78	27.32
185	20	10	44.64	10	70	30.00	15.36	270	210	100.35	27.7
186	20	10	38.45	10	70	30.00	21.55	270	240	100.47	27.53
187	20	10	30.00	10	70	30.00	30.00	270	270	100.63	27.42
188	20	10	15.36	10	70	30.00	44.64	270	300	100.72	27.32
189	20	10	30.00	10	70	44.64	15.36	300	210	100.53	27.71
190	20	10	23.81	10	70	44.64	21.55	300	240	100.61	27.58
191	20	10	15.36	10	70	44.64	30.00	300	270	100.68	27.39
192	20	10	54.64	40	40	15.36	20.00	210	90	100.72	27.76
193	20	10	46.19	40	40	15.36	28.45	210	120	100.7	27.86
194	20	10	40.00	40	40	15.36	34.64	210	150	100.43	27.85
195	20	10	29.28	40	40	15.36	45.36	210	210	100.54	27.88
196	20	10	23.09	40	40	15.36	51.55	210	240	101.18	28.07
197	20	10	14.64	40	40	15.36	60.00	210	270	101.49	28.15
198	20	10	48.45	40	40	21.55	20.00	240	90	100.82	27.78
199	20	10	40.00	40	40	21.55	28.45	240	120	100.8	27.89
200	20	10	33.81	40	40	21.55	34.64	240	150	100.52	27.86
201	20	10	23.09	40	40	21.55	45.36	240	210	100.62	27.88
202	20	10	16.91	40	40	21.55	51.55	240	240	101.38	28.4
203	20	10	40.00	40	40	30.00	20.00	270	90	100.92	27.8
204	20	10	31.55	40	40	30.00	28.45	270	120	100.89	27.89
205	20	10	25.36	40	40	30.00	34.64	270	150	100.6	27.86
206	20	10	14.64	40	40	30.00	45.36	270	210	100.69	27.84
207	20	10	25.36	40	40	44.64	20.00	300	90	101.13	27.85
208	20	10	16.91	40	40	44.64	28.45	300	120	101.13	27.92
209	20	10	10.72	40	40	44.64	34.64	300	150	100.86	27.87
210	20	10	39.28	70	10	15.36	35.36	210	60	101.38	27.47
211	20	10	24.64	70	10	15.36	50.00	210	90	101.74	27.97
212	20	10	16.19	70	10	15.36	58.45	210	120	101.48	28.18
213	20	10	10.00	70	10	15.36	64.64	210	150	100.71	27.99
214	20	10	33.09	70	10	21.55	35.36	240	60	101.5	27.51
215	20	10	18.45	70	10	21.55	50.00	240	90	101.85	28
216	20	10	10.00	70	10	21.55	58.45	240	120	101.6	28.21
217	20	10	24.64	70	10	30.00	35.36	270	60	101.59	27.53

218	20	10	10.00	70	10	30.00	50.00	270	90	102.01	28
219	20	10	10.00	70	10	44.64	35.36	300	60	101.92	27.53
220	20	40	54.64	10	40	20.00	15.36	90	210	100.65	27.56
221	20	40	48.45	10	40	20.00	21.55	90	240	100.81	27.38
222	20	40	40.00	10	40	20.00	30.00	90	270	101.05	27.26
223	20	40	25.36	10	40	20.00	44.64	90	300	101.31	27.29
224	20	40	46.19	10	40	28.45	15.36	120	210	100.74	27.83
225	20	40	40.00	10	40	28.45	21.55	120	240	100.9	27.71
226	20	40	31.55	10	40	28.45	30.00	120	270	101.13	27.6
227	20	40	16.91	10	40	28.45	44.64	120	300	101.31	27.72
228	20	40	40.00	10	40	34.64	15.36	150	210	100.45	27.8
229	20	40	33.81	10	40	34.64	21.55	150	240	100.58	27.68
230	20	40	25.36	10	40	34.64	30.00	150	270	100.76	27.61
231	20	40	10.72	10	40	34.64	44.64	150	300	100.97	27.76
232	20	40	29.28	10	40	45.36	15.36	210	210	100.41	27.75
233	20	40	23.09	10	40	45.36	21.55	210	240	100.51	27.64
234	20	40	14.64	10	40	45.36	30.00	210	270	100.55	27.48
235	20	40	23.09	10	40	51.55	15.36	240	210	100.91	27.84
236	20	40	16.91	10	40	51.55	21.55	240	240	100.93	27.64
237	20	40	14.64	10	40	60.00	15.36	270	210	101.13	27.67
238	20	40	50.00	40	10	20.00	20.00	90	90	100.9	27.24
239	20	40	41.55	40	10	20.00	28.45	90	120	100.9	27.35
240	20	40	35.36	40	10	20.00	34.64	90	150	100.77	27.54
241	20	40	24.64	40	10	20.00	45.36	90	210	101.11	28.08
242	20	40	18.45	40	10	20.00	51.55	90	240	101.74	28.4
243	20	40	10.00	40	10	20.00	60.00	90	270	102.04	28.6
244	20	40	41.55	40	10	28.45	20.00	120	90	101.11	27.63
245	20	40	33.09	40	10	28.45	28.45	120	120	101.07	27.73
246	20	40	26.91	40	10	28.45	34.64	120	150	100.88	27.84
247	20	40	16.19	40	10	28.45	45.36	120	210	101.18	28.22
248	20	40	10.00	40	10	28.45	51.55	120	240	101.83	28.59
249	20	40	35.36	40	10	34.64	20.00	150	90	100.82	27.63
250	20	40	26.91	40	10	34.64	28.45	150	120	100.81	27.75
251	20	40	20.72	40	10	34.64	34.64	150	150	100.57	27.78
252	20	40	10.00	40	10	34.64	45.36	150	210	100.88	28.13
253	20	40	24.64	40	10	45.36	20.00	210	90	101.02	27.9
254	20	40	16.19	40	10	45.36	28.45	210	120	101.01	28.02
255	20	40	10.00	40	10	45.36	34.64	210	150	100.71	27.99
256	20	40	18.45	40	10	51.55	20.00	240	90	101.57	28.06
257	20	40	10.00	40	10	51.55	28.45	240	120	102.6	28.24
258	20	40	10.00	40	10	60.00	20.00	270	90	101.91	28.12
259	20	70	39.28	10	10	35.36	15.36	60	210	101.19	28.07
260	20	70	33.09	10	10	35.36	21.55	60	240	101.33	27.99
261	20	70	24.64	10	10	35.36	30.00	60	270	101.52	28.01

262	20	70	10.00	10	10	35.36	44.64	60	300	101.7	28.22
263	20	70	24.64	10	10	50.00	15.36	90	210	101.72	28.55
264	20	70	18.45	10	10	50.00	21.55	90	240	101.85	28.52
265	20	70	10.00	10	10	50.00	30.00	90	270	101.99	28.6
266	20	70	16.19	10	10	58.45	15.36	120	210	101.67	28.55
267	20	70	10.00	10	10	58.45	21.55	120	240	101.8	28.58
268	20	70	10.00	10	10	64.64	15.36	150	210	100.88	28.12
269	30	10	23.92	10	40	18.04	18.04	210	210	99.3	26.44
270	30	10	14.64	10	40	18.04	27.32	210	240	99.38	26.38
271	30	10	14.64	10	40	27.32	18.04	240	210	99.36	26.42
272	30	10	31.96	40	10	18.04	10.00	210	90	99.59	26.51
273	30	10	19.28	40	10	18.04	22.68	210	120	99.72	26.59
274	30	10	10.00	40	10	18.04	31.96	210	150	99.53	26.58
275	30	10	22.68	40	10	27.32	10.00	240	90	99.68	26.51
276	30	10	10.00	40	10	27.32	22.68	240	120	99.83	26.59
277	30	10	10.00	40	10	40.00	10.00	270	90	99.85	26.52
278	30	40	31.96	10	10	10.00	18.04	90	210	99.57	26.46
279	30	40	22.68	10	10	10.00	27.32	90	240	99.69	26.38
280	30	40	10.00	10	10	10.00	40.00	90	270	99.86	26.52
281	30	40	19.28	10	10	22.68	18.04	120	210	99.78	26.7
282	30	40	10.00	10	10	22.68	27.32	120	240	99.9	26.72
283	30	40	10.00	10	10	31.96	18.04	150	210	99.6	26.64
